# Probing Electronic Doping in CVD Graphene Crystals Treated by HNO_3_ Vapors

**DOI:** 10.1021/acsomega.4c05697

**Published:** 2024-11-22

**Authors:** Nikos Delikoukos, Stavros Katsiaounis, John Parthenios, Labrini Sygellou, Dimitrios Tasis, Konstantinos Papagelis

**Affiliations:** 1 Institute of Chemical Engineering Sciences, Foundation of Research and Technology-Hellas (FORTH/ICE-HT), Stadiou Street, Platani, Patras 26504, Greece; 2 Department of Physics, University of Patras, Patras 26504, Greece; 3 Department of Chemistry, University of Ioannina, Ioannina 45110, Greece; 4 University Research Center of Ioannina (URCI), Institute of Materials Science and Computing, Ioannina 45110, Greece; 5 School of Physics, Department of Solid-State Physics, Aristotle University of Thessaloniki, Thessaloniki 54124, Greece

## Abstract

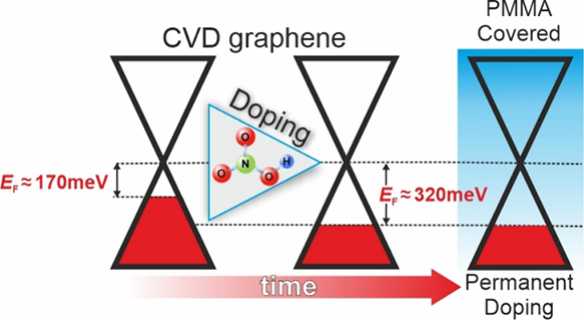

In this work, we present a comprehensive protocol for achieving hole doping in graphene through exposure to nitric acid (HNO_3_) vapors. We demonstrate gradual p-type surface doping of CVD-grown graphene on a Si/SiO_2_ substrate by thermally depositing nitric acid molecules to form self-assembled charge transfer complexes. Detailed analysis of charge carrier concentration and Fermi energy shifts was conducted using Raman, X-ray and ultraviolet photoelectron spectroscopies (XPS/UPS). Our methodology, including a novel PMMA coating step, ensures stability and efficiency of the doping process, highlighting its effectiveness in inducing permanent hole doping while maintaining the structural integrity of the graphene.

## Introduction

After extensive academic research, graphene has entered a phase of utilization in commercially available products like composite materials and sensors.^1^ As ongoing research progresses, the transition of graphene into an industrial-scale material is expected to be pivotal in the development of a wide range of products.^1,2^ In this context, the significant potential of graphene-based devices in fields like sensors,^3,4^ energy storage,^5,6^ and other electronic and optoelectronic devices^7,2,8^ makes these applications highly intriguing.^1,2^ Despite these promising advancements, the linear dispersed band structure and low carrier density of graphene are often considered limiting factors restricting the potential applications in various devices. Therefore, it is imperative to explore effective methods for controlling the charge carrier concentration and Fermi level of graphene.

Doping is a reliable approach for tailoring the electronic properties of graphene^9,10^ with numerous strategies outlined in the literature.^11^ These methods encompass chemical doping by substituting dopants in graphene lattice,^11,12^ physisorption of dopant molecules onto graphene,^10,11,13,14^ and electrostatic-field doping,^9,15^ all directing toward tuning the charge carrier density in graphene. Substitutional chemical doping offers advantages in terms of structural stability; however, it results in a significant reduction of charge carrier mobility primarily due to the introduction of defects in graphene’s honeycomb lattice.^16^ Consequently, the electrical properties undergo substantial degradation, posing challenges for the material’s use in electronic devices. For electrostatic-field doping, achieving electrostatic gating for millimeter-scale devices poses considerable challenges, prompting exploration of alternative gating and doping methods that do not necessitate a metallic top or bottom gate.^17,18^ Regarding physisorption, it is well known^19^ that graphene exhibits high sensitivity to surface dopants, due to its sp^2^-hybridized carbon atoms that readily react with the surrounding atmosphere.^19^ Through noncovalent anchoring, dopant molecules adhere to the graphene lattice, preserving its structural integrity and electrical properties.^20^ This approach offers a straightforward and efficient means of modifying graphene’s surface, inducing a significant change in carrier concentration of graphene, even on a large scale.

Unlike traditional semiconductors, the two-dimensional nature of graphene confines the doping process to surface adsorption. Heteroatom-containing systems including dyes, polymers, and fused aromatic systems have been employed to realize either *n*-type or *p*-type doping in the liquid phase.^14,21−23^ Unfortunately, these reactive molecules may introduce parallel doping effects, when utilizing liquid media for the doping step. In a representative work,^24^ researchers dipped graphene samples in aqueous mixtures of nitric acid at various concentrations. It was observed that the coadsorption of water molecules on the graphene surface led to a frequency shift of the G and 2D peaks in the Raman spectra. Additionally, immersing graphene samples in aqueous mixtures of nitric acid was found to potentially induce oxidative processes, as evidenced by an increase in peak D, due to the high acid concentration at the graphene surface. On the other hand, charge transfer doping using gases or volatile liquids has attracted significant interest due to its potential to avoid coadsorption phenomena.^13,25,26^ Specifically, gas-phase doping, such as with HNO_3_ vapors in our case, can effectively reduce secondary doping effects. Leveraging the undisturbed basal plane electron conjugation, gas-phase molecular charge transfer doping provides a facile and effective approach to dope graphene for future electronic devices.

In this work, we devised a strategy to achieve hole doping in graphene through exposure to nitric acid (HNO_3_) vapors. We demonstrate gradual *p*-type surface doping of supported (on Si/SiO_2_ substrate) graphene grown via chemical vapor deposition (CVD), a technique playing a pivotal role in graphene’s industrialization.^27,28^ Nitric acid molecules were thermally deposited to form self-assembled charge transfer complexes. The charge carrier concentration and shift of Fermi energy was meticulously analyzed using Raman, X-ray and ultraviolet photoelectron spectroscopies (XPS/UPS) for each doping step. Since the gas mixture was primarily nitric acid, the Fermi level shift during doping was mainly attributed to nitrogen molecules, as confirmed by UPS/XPS measurements. This combination of Raman, XPS and UPS techniques for the detailed physicochemical characterization of doped graphene introduces a methodology that has not been extensively explored or utilized in the field. A novel aspect of our method is the subsequent coating of the graphene surface with poly(methyl methacrylate) PMMA, which is a crucial step as it ensures stability and efficiency of hole doping in graphene. Our findings highlight the effectiveness of this method in inducing permanent hole doping in CVD graphene samples, while maintaining their structural integrity.

## Results and Discussion

**Figure 1:**
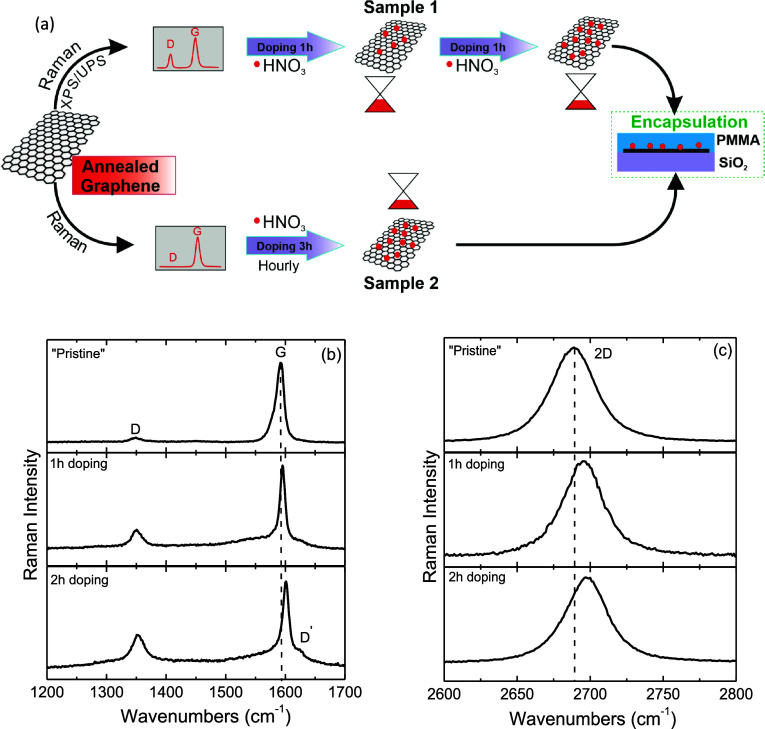
(a) Schematic illustration showing the doping/characterization sequence for two distinct approaches; Raman spectra of (b) the G peak and (c) the 2D peak before and after incremental exposure of *Sample 1* to nitric acid vapors.

Figure 1 outlines the doping and characterization sequence for the two approaches in this study. Initially, graphene is transferred onto a Si/SiO_2_ wafer and annealed at 295 °C to remove contaminants (see Methods for details). This “Pristine” graphene serves as the starting point for the doping process. Along the upper path of Figure 1a, the CVD graphene sample (hereafter referred to as *Sample 1*) is subjected to analysis employing both XPS/UPS and Raman spectroscopies. Concurrently, a parallel doping procedure is executed on another CVD graphene sample (*Sample 2*), exclusively investigated through Raman spectroscopy along the lower path of Figure 1a. Due to its sensitivity to structural and electronic characteristics of graphene, Raman spectroscopy has emerged as a valuable technique for investigating the influence of functional groups, determining doping states and type, and evaluating the shift of Fermi energy from the Dirac point.^29−31^ It is important to note that extensive Raman mappings (2600 spectra per step for each CVD graphene sample) were conducted to thoroughly investigate these aspects. Before analyzing the effects of the HNO_3_ treatment, it is important to note that the CVD graphene samples were fully characterized before and after annealing (see Section S1). As expected,^32^ the Raman spectra showed an upshift in the frequencies of the G and 2D peaks postannealing. The reasons for this blueshift in the main Raman peaks will be discussed later in the study.

Figure 1b and 1c depicts representative spectra of the G and 2D peaks before and after exposing *Sample 1* to nitric acid vapors, respectively. The spectra show noticeable shifts toward larger wavenumbers with doping. In the “Pristine” state of *Sample 1*, the G peak is positioned at 1589.2 ± 1.7 cm^–1^, with an average full width at half-maximum (FWHM) of 14.4 ± 2.1 cm^–1^. After 1 h of exposure to nitric acid vapors, the position of the G peak blueshifted to 1593.8 ± 1.6 cm^–1^, while the FWHM(G) decreased to 11.5 ± 1.5 cm^–1^. After an additional hour of exposure to nitric acid vapors (2 h in total), the G peak undergoes further blueshifting to 1598.7 ± 2.5 cm^–1^, while the FWHM(G) decreased even more to 10.2 ± 2.8 cm^–1^. The stiffening of the G mode with doping is attributed to the nonadiabatic removal of the Kohn anomaly at the Γ point of graphene’s Brillouin zone (BZ).^33^ Changing the Fermi energy of the system reduces the interaction between phonons and interband electron–hole pairs, which in turn affects the effective force constant of these atomic vibrations,^34^ where the sp^2^ carbon atoms move in-plane.^35^ For the same reason, the width of the G peak decreases with doping. This decrease eventually saturates when the electron–hole energy gap surpasses half of the phonon energy. Saturation occurs due to the suppression of phonon decay into electron–hole pairs (known as Landau damping) as a result of the Pauli exclusion principle.^9,34,36^ Additionally, the 2D Raman peak of *Sample 1* exhibits a similar albeit less pronounced behavior. In its “Pristine” state, it appears at 2692.7 ± 1.8 cm^–1^ with an FWHM(2D) of 33.3 ± 2.1 cm^–1^. After 1 and 2 h of doping, the position of the 2D peak blueshifted to 2695.4 ± 0.5 and 2696.4 ± 2.1 cm^–1^, respectively, while its FWHM slightly decreased (Table 1). It is well documented^9^ that the frequency position of the 2D peak shifts upward with *p*-type doping and downward with *n*-type doping. Unlike the G peak, nonadiabatic effects have a negligible impact on the 2D phonons since they are located away from the Kohn anomaly at the *K* point of graphene’s BZ. Therefore, the 2D peak position is primarily influenced by changes in the lattice constant.^9,34,36^Figure 1b also shows the presence of the D and D′ peaks at approximately 1350 and 1625 cm^–1^, respectively, which require the presence of defects for their activation. The increased intensity in these peaks with prolonged doping, as shown in Figure 1b, is attributed to UPS/XPS characterization rather than the doping process itself, as will be discussed further.

**Table 1:** Average Values and Standard Deviations of the Raman Spectral Features (G, D, and 2D Peaks) along with the Work Function Φ (eV) of *Sample 1*

Sample 1	Pos(G) (cm^–1^)	FWHM(G) (cm^–1^)	Pos(2D) (cm^–1^)	FWHM(2D) (cm^–1^)	Φ (eV)	*I*(D)/*I*(G)	*I*(2D)/*I*(G)
“Pristine”	1589.2 ± 1.7	14.4 ± 2.1	2692.7 ± 1.8	33.3 ± 2.1	4.12 ± 0.05	0.29 ± 0.10	7.3 ± 1.8
1 h doping	1593.8 ± 1.6	11.5 ± 1.5	2695.4 ± 0.5	30.6 ± 0.5		0.44 ± 0.10	6.1 ± 1.5
1 h doping/XPS-UPS	1589.7 ± 1.2	14.1 ± 1.6	2693.1 ± 1.4	31.8 ± 1.0	4.22 ± 0.05	0.56 ± 0.13	11.7 ± 2.5
2 h doping	1598.7 ± 2.5	10.2 ± 2.8	2696.4 ± 1.6	32.9 ± 0.9		0.60 ± 0.10	6.5 ± 2.6
2 h doping/XPS-UPS	1590.7 ± 2.2	14.3 ± 2.6	2693.0 ± 3.0	33.7 ± 2.8	4.31 ± 0.05	1.36 ± 0.14	7.1 ± 3.4

**Figure 2:**
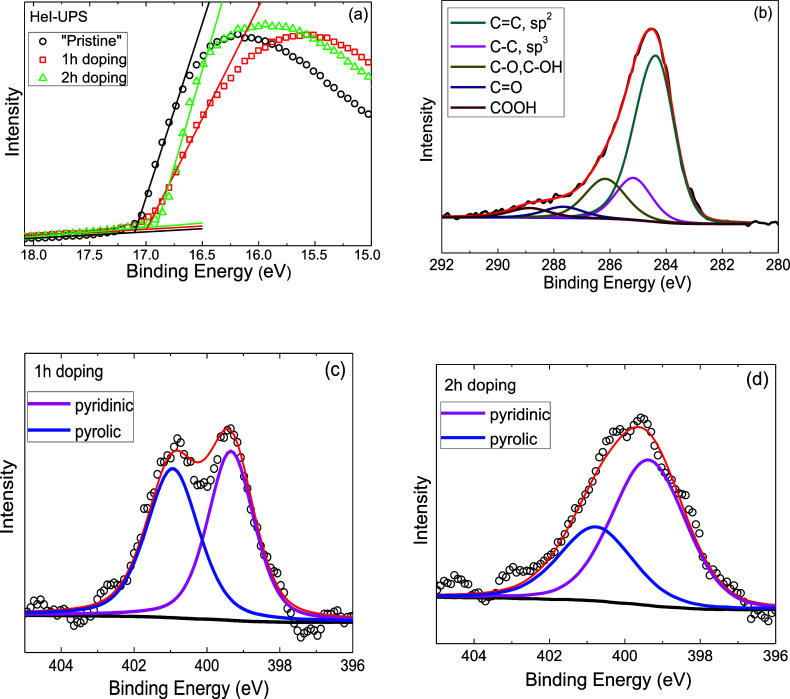
(a) High binding energy cutoff of the UPS spectra. The curves correspond to the “Pristine” material and after two doping levels (*Sample 1*). The arrows indicate the *E*_SEC_ (binding energy of the secondary electron cutoff), used to calculate the work function of the surfaces. (b) XPS spectrum of the C 1s peak of *Sample 1*. XPS spectra of the N 1s peak after (c) 1 h and (d) 2 h of doping with nitric acid vapors.

Following Raman analysis of *Sample 1*, surface chemical characterization was conducted using XPS/UPS, as outlined in the upper path of Figure 1a. Ultraviolet photoelectron spectroscopy was employed to thoroughly examine the work function (Φ) of *Sample 1* in its “Pristine” state and after each doping step, as presented in Figure 2a. From the figure, it can be observed that the *E*_SEC_ for the “Pristine” state is 17.10 eV, corresponding to a work function of 4.12 ± 0.05 eV. After 1 and 2 h of doping, the work function increases to 4.22 ± 0.05 and 4.31 ± 0.05 eV with *E*_SEC_ values of 17.00 and 16.91 eV, respectively. These results demonstrate a work function increase of 0.10 ± 0.05 eV after 1 h of exposure to nitric acid vapors and 0.19 ± 0.05 eV after 2 h, indicating p-type doping. To further investigate the nitrogen doping effect on the graphene surface, detailed core level spectra were recorded for both C 1s and N 1s, as illustrated in Figure 2. The presence of nitrogen on the surface is evident, indicating nitrogen adsorption after exposure to HNO_3_ vapors. The atomic N:C ratio, determined by the peak areas of N 1s and C 1s, weighted by the corresponding relative sensitivity factors (RSF) and the transmission function of the energy analyzer, was approximately 0.02 following each doping step, indicating a low concentration of nitrogen-containing moieties. In Figure 2b, the C 1s peak demonstrates a typical shape for the graphitic lattice, resolved into five components attributed to sp^2^ carbon and sp^3^ carbon at 284.4 and 285.2 eV, respectively, as well as various carbon–oxygen bonding configurations, including C–O bonds (at 286.4 eV), carbonyls (C=O) (at 287.7 eV), and carboxylates (O=C–O) (∼289 eV),^37^ which are present on the graphitic network due to air exposure prior to introduction into the UHV chamber for XPS/UPS measurements. The C 1s spectra of “Pristine” and doped samples were fitted with a mixed Gaussian–Lorentzian ratio after Shirley-type background subtraction, processed with XPS Spectra 4.1 software. An asymmetric Gaussian line shape (*T* = 0.15) with an FWHM of 1.6 eV was used for the C–C sp^2^ component, while a symmetric Gaussian line shape (FWHM = 1.9 eV) was applied for the C–C sp^3^ component. For oxygenated carbon species, symmetric mixed Gaussian–Lorentzian line shapes with equal widths (FWHM = 2.0 eV) and 40% Lorentzian contribution were used. The C–C sp^3^ component represents sp^3^ defects that appeared in the XPS C 1s spectra of the surfaces. The sp^3^/sp^2^ ratio is 0.20 in the pristine sample, and it increases to 0.27 and 0.29 after 1 and 2 h of doping, respectively, indicating an increase in defects within the graphene network. The N 1s spectra depicted in Figure 2c and 2d correspond to 1 and 2 h of doping processes, respectively. Despite the low signal-to-noise ratio of the N 1s spectra due to the low nitrogen concentration, the N 1s peak can be deconvoluted into two components. For 1 h doping (Figure 2c), a symmetric Gaussian–Lorentzian line shape with 40% Lorentzian contribution and FWHM 1.7 eV was used. For 2 h doping (Figure 2d), a symmetric Gaussian–Lorentzian line shape with 20–30% Lorentzian contribution and FWHM of 2.3 eV was applied. These components are attributed to nitrogen-based species with binding energies of 399.2 eV (pyridine-type) and 401.1 eV (pyrrolic-type).^12^ Under specific doping conditions, the formation of both five- and six-atom heteroatom-containing rings are expected to occur at the periphery or defect sites of the graphene lattice.^38^ However, the formation of chemically bound nitrogen species implying substitutional doping should be ruled out under the specific low-temperature conditions of the doping process.^39^ It is noteworthy that following each level of doping and subsequent UPS/XPS characterization, a red shift in the G and 2D Raman peak positions of graphene was observed (see Table 1). These shifts nearly approached those of the initial (“Pristine”) sample. We attribute this phenomenon to the ultrahigh vacuum conditions causing the desorption of loosely bound nitrogen-based species or even nitric acid molecules, as well as to X-ray irradiation. Sample degradation during X-ray measurements is a well-known phenomenon, especially for polymers,^40^ and has recently been studied in our UHV system on polymer-coated cotton fabrics, where the decrease of nitrogen and chloride ions during the X-ray exposure time was monitored.^41^ Consequently, in the present study, the UPS spectra were recorded first, followed by XPS, with the N 1s XPS peak measured initially.

By compiling the relevant vibrational parameters from the monitored regions of *Sample 1*, we could assess their correlation and progression with doping. Table 1 presents the mean values and corresponding standard deviations of the Raman spectral characteristics of the G and 2D peaks, along with the work function (eV), both before and after doping of *Sample 1*. Notably, the *I*(D)/*I*(G) ratio in Table 1, representing the integrated intensities of the D and G peaks (where *I* refers to peak area, not height, throughout the manuscript), suggests an increase in defects in *Sample 1* after UPS/XPS measurements. This is further corroborated by the elevated D band intensity in Figure 1c and the increased sp^3^/sp^2^ ratio observed in the XPS analysis. A similar behavior regarding the susceptibility of two-dimensional graphene in the presence of soft X-rays was documented by Zhou et al.^42^

**Figure 3:**
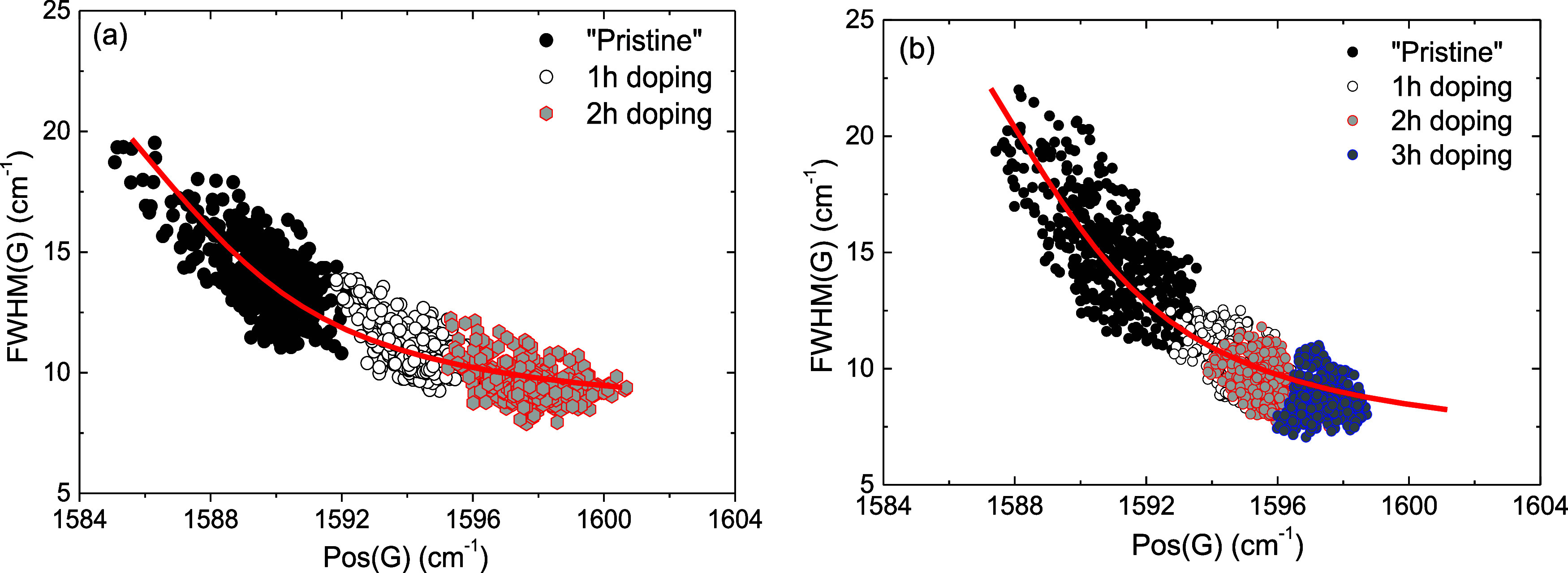
(a) Frequency position of the G peak (Pos(G)) as a function of FWHM(G) for (a) *Sample 1* and (b) *Sample 2*, both before and after the gradual chemical doping. Red lines serve as guide to the eye.

Figure 3 delineates the frequency position of the G band relative to its FWHM, both before and after each stage of gradual chemical doping, for *Samples 1* and 2. The red lines in the figure serve as a guide to the eye. As depicted in Figure 3a (and supported by the data in Table 1), exposing *Sample 1*, initially doped for 1 h, to nitric acid vapors for an additional hour resulted in a more pronounced blue shift of the G peak, accompanied by a further narrowing of its width. This indicates an enhancement in hole doping, attributed to the generation of surface defects. Consistently, a comparable but less pronounced trend was observed for the 2D peak as well (see Table 1 for details). In line with previous findings,^43^ it was reported that the presence of defects in graphene facilitated the adsorption of molecules of nitric acid onto its surface. Therefore, the existence of defects in our *Sample 1* heightened the concentration of adsorbed nitric acid molecules, leading to an additional shift in both the G and 2D peaks, thus reflecting the induction of hole doping. The *I(*2D)/*I*(G) ratio, representing the integrated intensities of the 2D and G peaks, is also presented in Table 1. It decreases with doping, consistent with other experimental observations.^9,26^ However, after UPS/XPS measurements, it tends to increase, as seen in Table 1, showing an unusual trend (*vide infra*).

To prevent desorption phenomena of nitrogen-based dopant species under ultrahigh vacuum conditions, we prepared another CVD graphene sample (*Sample 2*) and treated it with nitric acid vapors. Characterization was done exclusively through Raman spectroscopy (≈2600 spectra at each step), excluding XPS/UPS measurements. Figure 3b shows the G band’s frequency position relative to its width before and after each stage of gradual chemical doping over 3 h. As evident from Figure 3b, the characteristics of the G peak frequency are influenced by the presence of electronic doping similarly to *Sample 1*. However, the data in Figure 3b show a steeper trend for *Sample 2* because, as will be further explained, *Sample 2* experiences the largest shift in the Fermi level during the first hour of doping, with only minor changes thereafter. In contrast, *Sample 1* continues to exhibit changes in the Fermi level with prolonged doping due to the induction of point-like defects on the graphene surface, as observed from the UPS/XPS measurements and previously discussed.

Table 2 provides a comprehensive overview of the average values and corresponding errors for the Raman spectral features of the G and 2D peaks before and after the gradual doping of *Sample 2* with nitric acid vapors. In its “Pristine” state, *Sample 2* exhibits an average value of the frequency position of the G peak at 1590.8 ± 1.7 cm^–1^, with an FWHM(G) of 13.1 ± 1.3 cm^–1^. Following 1 h of exposure to nitric acid vapors, a noticeable shift is observed in the average G peak position to 1595.2 ± 0.9 cm^–1^, accompanied by a reduction in FWHM(G) to 10.0 ± 1.7 cm^–1^. Further doping for an additional hour (total duration 2 h) resulted in slight alterations of these parameters, with the values being 1596.5 ± 1.2 and 9.1 ± 1.5 cm^–1^, for Pos(G) and FWHM(G), respectively. Extending the doping duration for an additional hour (3 h in total), the average G peak frequency position centers at 1597.1 ± 1.1 cm^–1^, while the FWHM(G) is 8.9 ± 1.1 cm^–1^. The observed changes in the G peak frequency and FWHM(G) are mostly attributed to the shift of the Fermi energy (*E*_F_) away from the Dirac point, due to p-type doping. Notably, a diminishing shift of the G peak is apparent with prolonged exposure to nitric acid vapor, indicating saturation even after the initial 1 h doping period. This saturation suggests that a specific population of adsorbed nitric acid moieties reaches the maximum density of holes on the graphene surface within that time frame. A similar behavior is observed in the case of the 2D Raman peak, where its frequency position shifts toward higher wavenumbers after 1 h of doping but does not exhibit further blueshift for longer doping durations (Table 2). This behavior is consistent with our recent work,^26^ where we chemically doped CVD graphene samples with nitric acid vapors and observed a similar saturation effect after 1 h of treatment. Additionally, it is noteworthy that *Sample 2*, which did not undergo soft X-ray irradiation during XPS analysis like *Sample 1*, shows a relatively low defect density. This is evident by the comparatively low values (ranging from 0.09 ± 0.05 to 0.15 ± 0.1) of the *I*(D)/*I*(G) ratios at each step, as presented in Table 2, and the absence of the D′ peak. This observation further supports the idea that the specific doping process itself does not appear to induce defects on the graphene surface. This finding is also consistent with our previous work^26^ and underscores that the defects observed in *Sample 1* were likely generated during the XPS/UPS procedure. Moreover, as evident from Table 2, the *I*(2D)/*I*(G) ratio which is another indicator of doping in graphene,^9,26,34^ decreases after 1 h of doping, as expected,^9,26^ and then remains relatively constant. Representative Raman spectra of *Sample 2* in the frequency region of the G and 2D peaks before and after gradual exposure to nitric acid are shown in Figure S2.

**Table 2:** Average Values and Their Corresponding Errors for the Raman Spectral Characteristics (G, D, and 2D Peaks) of *Sample 2*

Sample 2	Pos(G) (cm^–1^)	FWHM(G) (cm^–1^)	Pos(2D) (cm^–1^)	FWHM(2D) (cm^–1^)	*I*(D)/*I*(G)	*I*(2D)/*I*(G)
“Pristine”	1590.8 ± 1.7	13.1 ± 1.3	2692.3 ± 1.3	32.8 ± 1.1	0.09 ± 0.05	5.6 ± 1.3
1 h doping	1595.2 ± 0.9	10.0 ± 1.7	2695.7 ± 1.3	29.9 ± 1.2	0.1 ± 0.08	4.4 ± 0.9
2 h doping	1596.5 ± 1.2	9.1 ± 1.5	2693.8 ± 1.8	30.3 ± 3.0	0.15 ± 0.09	4.1 ± 1.0
3 h doping	1597.1 ± 1.1	8.9 ± 1.1	2694.9 ± 1.5	30.6 ± 3.2	0.15 ± 0.1	4.3 ± 1.7

To explore the potential for further shifts in the Raman peaks, we exposed *Sample 2* to an additional hour in nitric acid vapors, increasing the total exposure time to 4 h. Unfortunately, this extended exposure caused significant damage to the graphene sample, leading to numerous folds and cracks in its lattice, as shown in the optical microscope image in Figure S3. As a result, further Raman analysis of *Sample 2* is deemed unnecessary as it would not provide additional insights for this research.

Our attention now shifts to the determination of carrier concentration and the shift of *E*_F_ in the CVD graphene samples. Initially, in *Sample 1*, the experimental calculation of the work function is conducted both before and after the doping process using UPS (see Table 1). The results revealed a Fermi level shift of approximately 200 meV compared to the “Pristine” state. Another approach to determine the shift of *E*_F_ involves using Raman spectroscopy measurements. When transferring CVD graphene onto the desired substrate, residual doping and biaxial mechanical strain are typically present.^26^ Furthermore, during the doping process, it is likely that additional mechanical strain will be introduced alongside an increase in carrier concentration.^26^ Raman spectroscopy has proven effective in differentiating strain and doping effects in graphene by examining the correlation between the Raman shifts of the G and 2D bands.^44,45^

At this point, we must first address the analysis of Raman parameters for the CVD graphene samples before and after annealing (referred to as the “Pristine” state in this manuscript). Data in Tables S1 and S2 show an upward shift in the G and 2D peak positions postannealing, indicating crystal lattice compression due to high temperature, with strain increasing from about 0.07 to 0.12%. Table S1 also confirms no additional hole doping from exposure to ambient O_2_ molecules during annealing, as evidenced by the slight reduction in the Fermi level. This suggests that the residual doping primarily originated from the transfer process. Additionally, Tables S4 and S6 show that compressive strain increased to 0.13–0.18% after the doping process.

Tables 3 and 4 present the calculated values of *n*_h_ and *E*_F_ (see Methods for details) for *Samples 1* and 2 in each doping step, respectively. As shown in Table 3, *Sample 1* exhibits unintentional doping during fabrication, with its Fermi energy positioned 149 ± 40 meV below the Dirac point in its “Pristine” state. After 1 h of doping with nitric acid vapors, the calculated *E*_F_ value was 245 ± 26 meV, reflecting a shift of ≈100 meV. Following 2 h of doping, *E*_F_ shifted further by ≈180 meV, reaching a value of 328 ± 20 meV. These values closely match the estimates obtained from UPS, where the corresponding *E*_F_ shifts for 1 and 2 h of doping were 100 and 190 meV, respectively. This coincidence underscores the ability of Raman spectroscopy in determining the carrier concentration in graphene samples. Moreover, as shown in Table 4, *Sample 2* exhibits residual doping from the fabrication process, with *E*_F_ calculated to be 199 ± 32 meV below the Dirac point in its “Pristine” state. After 3 h of doping, *E*_F_ shifts by approximately 110 meV, resulting in a value of 312 ± 21 meV. However, the most significant shift in *E*_F_ occurs during the initial hour of doping, as shown in Table 4. After 2 h of exposure to nitric acid vapors, *Sample 2* experiences a modest increase in doping, with further doping having a limited effect after 3 h. Additionally, as previously mentioned, the larger *E*_F_ shifts observed in *Sample 1* compared to *Sample 2* after each doping step, as indicated in Tables 3 and 4, can be attributed to the higher level of induced defects in *Sample 1* resulting from UPS/XPS. This is supported by Raman spectroscopy through the *I*(D)/*I*(G) ratio in Table 1. These defects likely facilitate the adsorption of additional nitric acid molecules on the surface of *Sample 1*.^43^

**Table 3:** Average Hole Concentration and the Mean Shift in Fermi Energy Resulting from Electronic Doping of *Sample 1*, Derived from the Raman Spectroscopic Parameters

Sample 1	“Pristine”	1 h doping	after XPS-UPS	2 h doping	after XPS-UPS
*n*_h_ (× 10^13^ cm^–2^)	0.17 ± 0.08	0.44 ± 0.06	0.22 ± 0.07	0.79 ± 0.05	0.29 ± 0.07
Δ*E*_F_ (meV)	149 ± 40	245 ± 26	173 ± 36	328 ± 20	199 ± 32

**Table 4:** Average Hole Concentration and the Mean Shift in Fermi Energy Resulting from Electronic Doping of *Sample 2*, Derived from the Raman Spectroscopic Parameters

*Sample 2*	“Pristine”	1 h doping	2 h doping	3 h doping
*n*_h_ (× 10^13^ cm^–2^)	0.29 ± 0.07	0.54 ± 0.06	0.71 ± 0.05	0.71 ± 0.05
Δ*E*_F_ (meV)	199 ± 32	270 ± 24	310 ± 21	312 ± 21

The main drawback of doping using gaseous species lies in the system’s instability due to reversible adsorption and desorption processes.^11^ To overcome this limitation and maintain the induced hole doping on the graphene surface, we adopted a strategy of covering the supported nanostructure with a thin PMMA film. As previously discussed, after UPS/XPS measurements of *Sample 1*, we observed the desorption of nitric acid molecules from its surface, causing the Fermi energy to revert close to its “Pristine” state (see Table 1). Therefore, before applying the PMMA coating to *Sample 1*, we exposed it to an additional 2 h of nitric acid vapors to restore the initial 2 h doping prior to UPS/XPS measurements. To validate the success of this process, we conducted extensive Raman measurements (≈2600 spectra) on *Sample 1* immediately after its 2 h exposure to nitric acid vapors, before the PMMA coating (refer to Table S3). From these Raman findings, the Fermi energy shift was determined to be 302 ± 21 meV (≈0.67 × 10^13^ cm^–2^), closely aligning with the 328 ± 20 meV (≈0.79 × 10^13^ cm^–2^) shift observed after the initial 2 h doping before UPS/XPS analysis (refer to Table 1). Subsequently, immediately after its PMMA coating and again after 1 month, we conducted Raman measurements (≈2600 spectra/step) on *Sample 1* to investigate whether the doping level is retained. The minor changes in the average positions of G and 2D peaks, immediately after the sample coating, are attributed to a slight desorption of nitric acid molecules from its lattice as evident from Table S3. Additionally, Table S4 shows that some extra mechanical strain was introduced in *Sample 1* following the PMMA coating. As also evident from Table S3, after 30 days of the polymer-coated *Sample 1*, the level of electronic doping remained consistent. This analysis underscores that coating chemically doped graphene with a thin PMMA layer effectively preserves the adsorbed nitric acid molecules on the graphene surface, ensuring long-term stability while maintaining structural integrity.

A similar treatment was also applied to another supported graphene sample, which did not undergo surface characterization via XPS. As mentioned earlier, we aimed to explore the potential for further shifting of the G peak and consequently increasing the induced electronic doping; *Sample 2* was exposed to nitric acid vapors for 4 h, resulting in severe structural damage. Subsequently, a newly prepared supported graphene sample (referred to as *Sample 3*) was subjected to 3 h of continuous exposure to nitric acid vapors to approximate the state of *Sample 2* after 3 h of doping as closely as possible (characteristic Raman plots of *Sample 3* are presented in Figure S4). Tables S5 and S6 present the average values and their corresponding errors of the Raman spectral characteristics of *Sample 3* after 3 h of exposure to nitric acid vapors, both before and after being covered with a thin PMMA film, as well as after a 30-day period from the application of the film (≈2600 Raman spectra/step). As indicated in Table S5, the Fermi energy shift for *Sample 3* (302 ± 23 meV) closely corresponds to that of *Sample 2* (312 ± 21 meV) after 3 h of doping (also see Table 2). Following the application of PMMA coating to *Sample 3*, minor shifts in the average positions of the G and 2D peaks were observed (Table S5), indicating minimal changes in the doping level and a slight reduction in mechanical strain (refer to Table S6) compared to the “Pristine” state. After a 30-day period, no significant alterations were detected, as evident in Table S6, confirming the maintenance of electronic doping after coating *Sample 3* with PMMA. This highlights the effectiveness of this method in preventing the desorption of absorbed nitric acid molecules from the graphene surface, akin to the observations with *Sample 1*.

## Conclusions

We conducted gradual *p*-type surface doping of supported CVD graphene samples with thermally deposited HNO_3_ molecules, achieving a maximum Fermi energy shift by approximately 320 meV from the Dirac point. Detailed analysis of the charge transfer mechanism was performed using Raman, X-ray and Ultraviolet photoelectron spectroscopies (XPS/UPS) for each doping step, a combination of techniques not previously reported. Our observations revealed that XPS/UPS measurements triggered the desorption of loosely bound nitric acid molecules from the graphene surface. Additionally, it was shown that UPS/XPS induce defects in graphene lattice, as verified by the increased *I*(D)/*I*(G) ratio. These surface defects enhanced hole doping after subsequent doping steps following UPS/XPS measurements. Raman spectroscopy alone showed saturation of doping effects after just 1 h of doping, with relatively low defect density, confirming that the specific doping process itself does not induce defects. To address the challenges of adsorption and desorption associated with gaseous doping, we employ a strategy of coating the supported CVD graphene samples with a thin PMMA film. This approach effectively maintains permanent electronic doping in graphene, which is crucial for its application in various electronic devices.

## Methods

### Fabrication of CVD Graphene Samples

The samples, synthesized using the CVD method on polycrystalline copper foils, were provided by AIXTRON. We used a standard wet transfer method for sample transfer.^46,47^ A PMMA layer was spin-coated at 600 rpm for 2 min in air, forming a thin dried PMMA film over the CVD graphene, with no additional drying required. Subsequently, they were immersed in a NaOH solution (25 mL of deionized water with 1 mg of NaOH) for 3 h to dissolve the copper substrate. Once the copper was dissolved, the PMMA film with the graphene was transferred to a container of deionized water and was repeatedly cleaned to ensure complete removal of the etchant, leaving the film floating in the DI water. Following this step, the films were transferred onto the 300 nm Si/SiO_2_ substrate wafers and then left to dry overnight in an inert atmosphere. Finally, to remove the PMMA film, the Si/SiO_2_/CVD graphene/PMMA sample was repeatedly washed using hot acetone followed by thermal annealing at 295 ± 5 °C for 15 min. The successful removal of the PMMA was confirmed through optical microscopy images.

### Doping Method

A straightforward and highly efficient method for doping CVD graphene using HNO_3_ vapors (65% w/v) has been devised, eliminating the need for liquid-phase protocols. After transferring the graphene samples to Si/SiO_2_ substrates, they were positioned above a concentrated HNO_3_ solution heated to 120 °C (approximately the boiling point of nitric acid) to ensure smooth evaporation. The temperature in the sample area was maintained at 75–80 °C using hot air to prevent condensation of HNO_3_ molecules. The vapor phase consisted primarily of HNO_3_, along with O_2_ and H_2_O. The nitric acid molecules were physically adsorbed onto the surface without forming chemical bonds with the graphene, resulting in *p*-type doping.

### Raman Spectroscopy

Spectra were acquired employing a MicroRaman spectrometer (InVia 2000, Renishaw, UK) with a 514.5 nm (2.41 eV) laser. The instrument offers approximately 2 cm^–1^ spectral resolution and 0.1 cm^–1^ spectral accuracy. To prevent laser-induced local heating, the laser power was maintained below 1.5 mW on the sample. An Olympus MPLN100× objective (NA = 0.90) was employed to focus the beam onto the samples. Raman mapping was conducted within a rectangular area measuring 50 × 50 μm, utilizing a high-speed optically encoded motorized sample stage (Renishaw, UK) with a step size of 1 μm. The spectral line shape parameters were obtained by fitting Lorentzian functions to the experimental peaks following background subtraction. The analysis of the Raman spectral characteristics of the pristine CVD-grown graphene samples indicates high structural quality, with no significant variations in the Raman spectral features across different samples and sample areas.

### Determination of Hole Concentration via Raman Spectroscopy

The estimation of hole concentration (as nitric acid induces p-type doping) based on the G band position involved fitting the data from ref (9) using a quadratic polynomial equation, given by
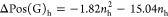
1where *n*_h_ is the concentration of holes in 10^13^ cm^–2^ and ΔPos(*G*)_h_ is the shift of the G peak due to doping alone. ΔPos(G)_h_ is determined by the relation
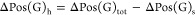
2Here, the term ΔPos(G)_tot_ represents the total shift of the measured G peak from the corresponding value of free-standing graphene (1581 cm^–1^).^48,49^ The ΔPos(G)_s_ is the shift of the G band due to pure mechanical strain. It is evident from ref (9) that Pos(2D) remains constant upon doping, for *n*_h_ up to approximately 0.5 × 10^13^ cm^–2^. Thus, the deviation of the measured Pos(2D) from the value of free-standing graphene (2680 cm^–1^)^48,49^ in “Pristine” samples is solely attributed to mechanically induced strain. In experiments involving pure biaxial loading, it was demonstrated that the shift ratio of the G and 2D bands, (∂Pos(2D)/∂Pos(G))_s_, is approximately 2.2 for both tensile and compressive strains.^44,50^ Considering the above, we can extract the value of Pos(G)_S_, which corresponds to mechanical strain alone by the relation
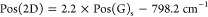
3

Thus, from eq 3, we can derive ΔPos(G)_s_ = Pos(G)_s_ – 1581 and from eq 2, we calculate ΔPos(G)_h_.^45^ Consequently, *n*_h_ is determined from eq 1, and as a final step, the change of *E*_F_ can be calculated by the equation,  ; |*v*_f_| is the absolute value of Fermi velocity (1 × 10^6^ m· s^–1^).^9^

### X-ray and Ultraviolet Photoelectron Spectroscopies

X-ray and ultraviolet photoelectron spectroscopy (UPS/XPS) measurements were performed in a UHV chamber (*P* ∼ 5 × 10^–10^ mbar) equipped with a SPECS EA10 hemispherical electron analyzer, a dual-anode (Mg/Al) X-ray gun, and a UV source (model UVS 10/35). XPS was recorded using Mg Kα radiation with a photon energy of 1253.6 eV and an analyzer pass energy of 36 eV, resulting in an FWHM of 1.0 eV for the Ag 3d5/2 line. UPS spectra were recorded using HeI irradiation with *h*ν = 21.22 eV, with the analyzer operating in the constant retarding ratio (CRR) mode, where CRR = 10. The instrument’s energy resolution is 0.5 eV, measured from the Fermi edge of Au foil, and the energy axis of the spectra was calibrated using the Au Fermi level, where the binding energy equals zero. A bias of −12.30 V was applied to the sample to avoid interference from the spectrometer threshold in the UPS spectra. The work function was estimated using the equation WF = *h*ν – (*E*_SEC_ – *E*_F_), where WF, *E*_SEC_, and *E*_F_ are the work function, secondary electron cutoff, and Fermi level of the spectrometer, respectively, and *h*ν is the photon energy of HeI (21.22 eV).^51^ The Fermi levels of the sample surface and sample holder (which is the same as that of the spectrometer) are aligned and are set at 0 eV. Since our samples are conductive, the work function of the surface is given by WF = *h*ν – *E*_SEC_. *E*_SEC_ was determined by linear extrapolation to the background, in the HeI UPS spectrum plotted with respect to the Fermi level as indicated by arrows in Figure 2a. The XPS survey scan confirms the presence of C, O, and Si elements. The spectra were fitted after Shirley background subtraction using the XPSPEAK 4.1 software. The photoelectron binding energy (BE) is referenced to the Fermi level of the analyzer and is reported as measured, with no additional corrections applied.

## Supporting Information Available

Characteristic Raman spectra and spectral parameters of CVD graphene samples before and after annealing; characteristic Raman spectra of *Sample 2* in each step*;* optical images of *Sample 2* before and after the doping process; Raman spectral parameters of *Sample 1* before and after its coverage with PMMA; characteristic Raman spectra and Raman spectral parameters of *Sample 2*; characteristic Raman spectra and Raman spectral parameters of *Sample 3* before and after its coverage with PMMA; and characteristic Raman spectra of “pristine” and doped CVD graphene (PDF)
