# Supplementary material for: Probing Electronic Doping in CVD Graphene Crystals Treated by HNO3 Vapors
Source: ACS Omega. 2024 Nov 22;9(49):48246–55. doi: 10.1021/acsomega.4c05697 (PMC11635467; doi:10.1021/acsomega.4c05697)
Supplement: Supplementary file 1 — ao4c05697_si_001.pdf [file ao4c05697_si_001.pdf]

Probing electronic doping in CVD graphene crystals treated by HNO_3 vapors

Nikos Delikoukos[1,2], Stavros Katsiaounis[1], John Parthenios[1], Labrini Sygellou[1], Dimitrios Tasis[3,4], and Konstantinos Papagelis[1,5*]*

[1]*Institute of Chemical Engineering Sciences, Foundation of Research and Technology-Hellas (FORTH/ICE-HT), Stadiou Street, Platani, Patras, 26504 Greece*

[2]*Department of Physics, University of Patras, Patras, 26504, Greece*

[3]*Department of Chemistry, University of Ioannina, 45110 Ioannina, Greece*

[4]*University Research Center of Ioannina (URCI), Institute of Materials Science and Computing, Ioannina 45110, Greece*

[5]*School of Physics, Department of Solid-State Physics, Aristotle University of Thessaloniki, Thessaloniki 54124, Greece*

Contents

S1. Characteristic Raman spectra and Spectral Parameters of CVD graphene samples before and after annealing. 3

S2. Characteristic Raman spectra of Sample 2 in each step 6

S3. Optical images of Sample 2 before and after doping process. 7

S4. Raman spectral parameters of Sample 1 before and after its coverage with PMMA. 8

S5. Characteristic Raman spectra and Raman spectral parameters of Sample 2 9

S6. Characteristic Raman spectra and Raman spectral parameters of Sample 3 before and after its coverage with PMMA. 9

S7. Characteristic Raman spectra of "Pristine" and doped CVD graphene 11

S1. Characteristic Raman spectra and Spectral Parameters of CVD graphene samples before and after annealing.

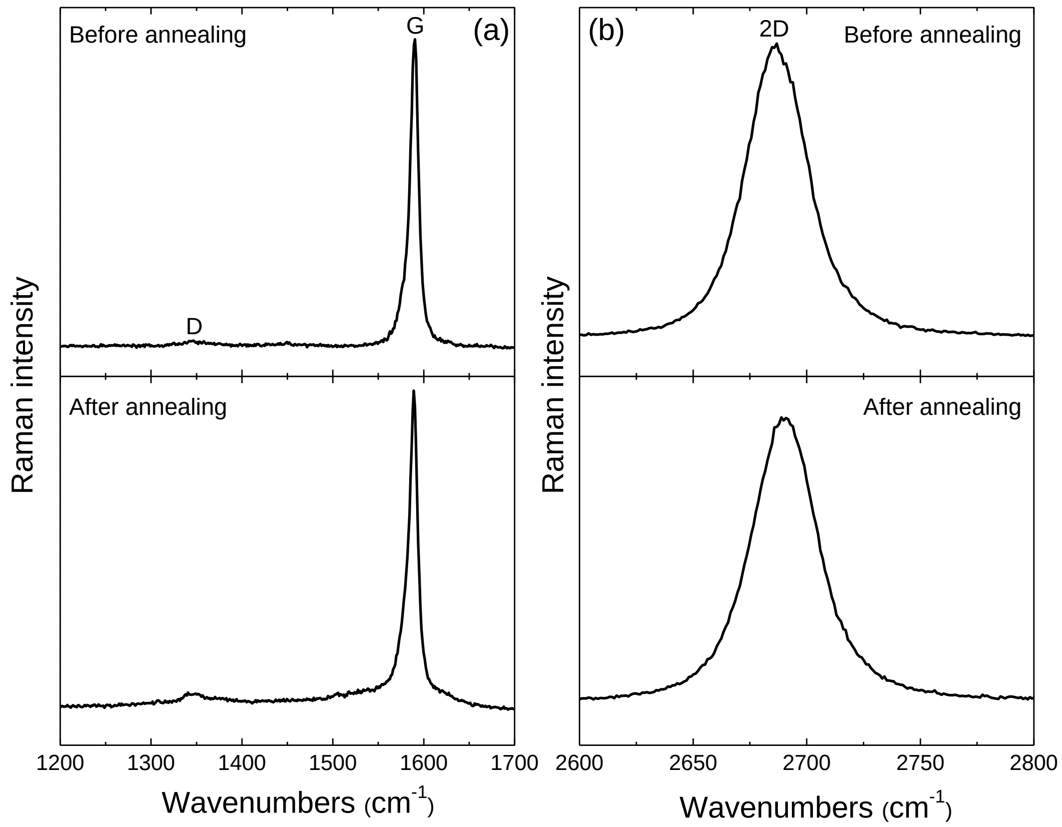

Figure S1. Representative Raman spectra of G (a) and 2D (b) peaks before and after annealing of the CVD graphene samples.

Table S1. Mean and standard deviation values of the Raman spectral features (G, D and 2D peaks) for *CVD graphene samples* both before and after annealing.

	Pos (G) (cm^{-1})	Pos (2D) (cm^{-1})	FWHM (G) (cm^{-1})	FWHM (2D) (cm^{-1})	I(D)/I(G)	I(2D)/I(G)	ΔE_F (meV)	n_h (x $10^{13}cm^{-2}$)
				Sample 1				
Before annealing	1588.1±2.3	2686.6±3.0	15.2±1.6	31.7±2.5	0.13±0.01	7.5±1.5	196±54	0.28±0.08
After annealing	1589.2±1.7	2692.7±1.8	14.4±2.1	33.3±2.1	0.29±0.1	7.3±1.8	149±40	0.17±0.08
				Sample 2				
Before annealing	1589.0±1.5	2687.9±1.9	12.1±1.8	30.6±2.2	0.08±0.03	5.9±1.4	224±34	0.31±0.06
After annealing	1590.8±1.7	2692.3±1.3	13.1±1.3	32.8±1.1	0.09±0.05	5.6±1.3	199±32	0.29±0.07
				Sample 3				
Before annealing	1588.1±1.1	2686.7±1.2	14.0±2.2	32.1±1.4	0.18±0.08	6.6±2.0	195±54	0.28±0.07
After annealing	1589.7±1.6	2691.3±1.6	15.1±2.8	33.5±1.7	0.14±0.09	6.1±0.4	183±32	0.24±0.05

It is evident from Table S1 that the *I(D)/I(G)* ratio for *Samples 2* and *3* remains unaffected by the annealing process, while for *Sample 1*, it increases from 0.13 to 0.29. This inconsistency arises because Raman characterization of *Sample 1* in its "pristine" state was performed after the UPS/XPS analysis, which, as explained in the manuscript, induces defects in the graphene lattice.

Table S2. Mean and standard deviation values of the Raman spectral features (D and D′ peaks) for *CVD graphene samples* both before and after annealing.

	Pos (D) (cm^{-1})	Pos (D′) (cm^{-1})	FWHM (D) (cm^{-1})	FWHM (D′) (cm^{-1})	*I*(D)/*I*(D′)	*Strain* (%)
			Sample 1			
Before annealing	1347.5±1.6	–	21.6±2.0	–	–	0.07
After annealing	1348.7±0.9	1626.9±1.0	20.5±1.1	10.8±1.3	12.4±1.8	0.13
			Sample 2			
Before annealing	1346.8±1.5	–	21.9±3.0	–	–	0.08
After annealing	1348.3±1.6	–	28.4±4.7	–	–	0.12
			Sample 3			
Before annealing	1346.1±1.0	–	22.1±1.9	–	–	0.07
After annealing	1347.8±1.3	–	26.7±4.2	–	–	0.11

S2. Characteristic Raman spectra of Sample 2 in each step

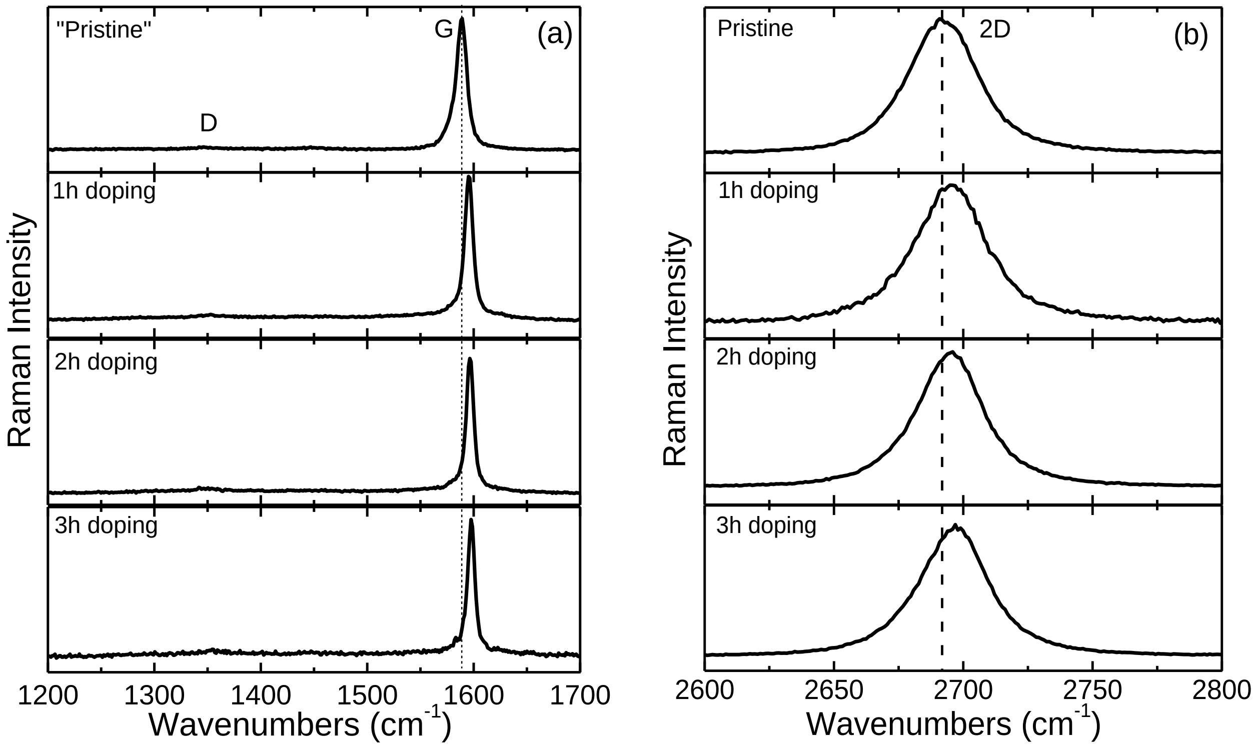

Figure S2. Raman spectra of G (a) and 2D (b) peaks before and after gradual exposure of *Sample 2* to nitric acid vapors.

S3. Optical images of Sample 2 before and after doping process.

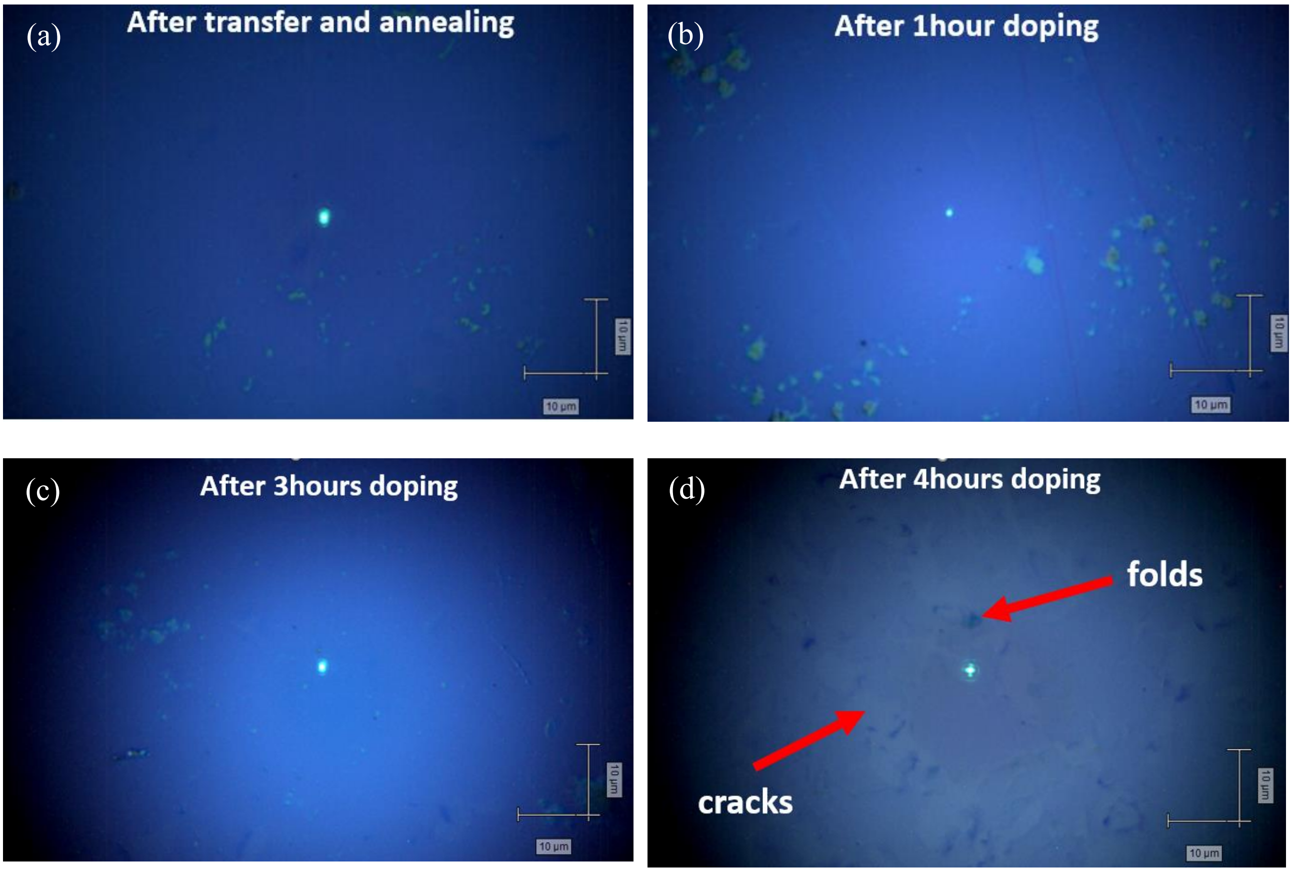

Figure S3. Optical microscope images of CVD graphene *Sample 2* a) before, b) after one, c) after three and d) after four hours of doping with nitric acid vapors.

S4. Raman spectral parameters of Sample 1 before and after its coverage with PMMA.

Table S3. Mean and standard deviation values of the Raman spectral features (G, D and 2D peaks) and calculated Fermi level (and holes concentration) for *Sample 1* both before and after its coating with PMMA, and also for a 30-days period post-coating.

Sample 1	Pos (G) (cm^{-1})	Pos (2D) (cm^{-1})	FWHM (G) (cm^{-1})	FWHM (2D) (cm^{-1})	I(D)/I(G)	I(2D)/I(G)	ΔE_F (meV)	n_h (x $10^{13} cm^{-2}$)
After 2 h doping	1597.8±1.8	2697.6±1.8	9.7±1.4	32.5±2.0	1.0±0.5	5.9±1.4	302±21	0.67±0.05
After PMMA coating	1596.5±2.2	2696.9±2.3	10.2±1.8	32.7±2.1	1.10±0.90	6.0±1.6	285±23	0.59±0.06
30-days post-coating	1596.4±1.5	2696.7±1.8	11.1±2.0	33.1±2.5	1.15±1.26	5.3±1.5	284±23	0.59±0.06

Table S4. Mean and standard deviation values of the Raman spectral features (D and D´ peaks) and calculated strain for *Sample 1*.

Sample 1	Pos(D) (cm^{-1})	FWHM(D) (cm^{-1})	Pos(D´) (cm^{-1})	FWHM(D´) (cm^{-1})	I(D)/I(D´)	*Strain* (%)
"Pristine"	1348.7±0.9	20.5±1.1	1626.9±1.0	10.8±1.3	12.4±1.8	0.13
1 h doping	1350.0±0.5	20.4±1.5	1626.7±0.7	11.1±2.2	9.2±2.6	0.15
1 h doping/XPS-UPS	1349.4±0.5	20.2±1.7	1627.3±0.9	12.4±1.9	9.5±1.6	0.12
2 h doping	1349.9±0.7	22.2±1.3	1627.1±0.9	10.8±1.7	9.9±2.4	0.16
2 h doping/XPS-UPS	1349.2±1.4	20.9±1.5	1626.7±1.4	12.2±2.2	11.5±1.9	0.12
2 h doping (repeat)	1351.7±1.0	21.5±1.6	1626.9±0.9	11.2±2.8	10.5±2.5	0.13
After PMMA coating	1351.4±2.1	22.4±2.7	1626.8±1.3	12.5±1.8	9.3±2.3	0.17
30-days post-coating	1351.1±1.0	22.3±1.6	1627.3±1.1	12.1±1.2	10.4±1.6	0.17

S5. Characteristic Raman spectra and Raman spectral parameters of Sample 2

Table S5. Mean and standard deviation values of the Raman D peak and calculated strain for *Sample 2*.

Sample 2	Pos(D) (cm^{-1})	FWHM(D) (cm^{-1})	*Strain* (%)
"Pristine"	1348.3±1.6	28.4±4.7	0.12
1 h doping	1350.1±1.3	27.4±4.3	0.15
2 h doping	1349.1±1.51	29.9±5.4	0.13
3 h doping	1350.8±1.5	29.8±5.6	0.14

S6. Characteristic Raman spectra and Raman spectral parameters of Sample 3 before and after its coverage with PMMA.

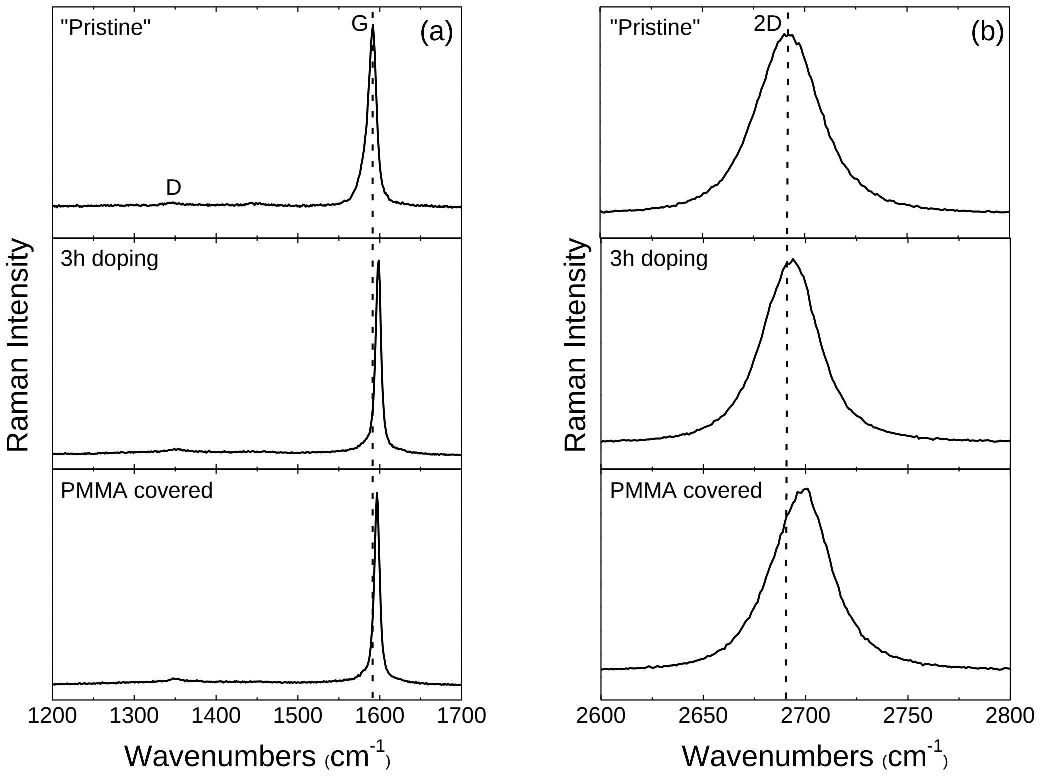

Figure S4. Raman spectra of G (a) and 2D (b) peaks before and after 3 hours exposure of *Sample 3* to nitric acid vapors as well as after its coverage with PMMA.

Table S6. Mean and standard deviation values of the Raman spectral features (G, D and 2D peaks) for *Sample 3* both before and after its coating with PMMA, and also for a 30-day period post-coating.

Sample 3	Pos (G) (cm^{-1})	Pos (2D) (cm^{-1})	FWHM (G) (cm^{-1})	FWHM (2D) (cm^{-1})	I(D)/I(G)	I(2D)/I(G)	ΔE_F (meV)	n_h (x $10^{13}cm^{-2}$)
"Pristine"	1589.7±1.6	2691.3±1.6	15.1±2.8	33.5±1.7	0.14±0.09	6.1±0.4	183±32	0.24±0.05
After 3h doping	1597.7±0.8	2697.4±1.1	8.8±1.5	30.3±1.0	0.20±0.11	4.9±0.9	302±23	0.67±0.06
After PMMA coating	1596.9±1.7	2696.2±2.0	9.2±1.2	29.4±1.7	0.28±0.15	5.8±0.9	298±22	0.65±0.05
30-days post-coating	1596.6±1.0	2695.9±1.4	9.3±1.7	30.2±1.2	0.26±0.11	5.7±0.7	295±22	0.64±0.05

Table S7. Mean and standard deviation values of the Raman D peak and calculated strain for *Sample 3*.

Sample 3	Pos(D) (cm^{-1})	FWHM(D) (cm^{-1})	*Strain* (%)
"Pristine"	1347.8±1.3	26.7±4.2	0.11
3 h doping	1349.9±2.7	24.2±3.6	0.18
After PMMA coating	1348.9±1.0	25±3.2	0.16
30-days post-coating	1347.9±1.0	25±3.1	0.16

S7. Characteristic Raman spectra of "Pristine" and doped CVD graphene

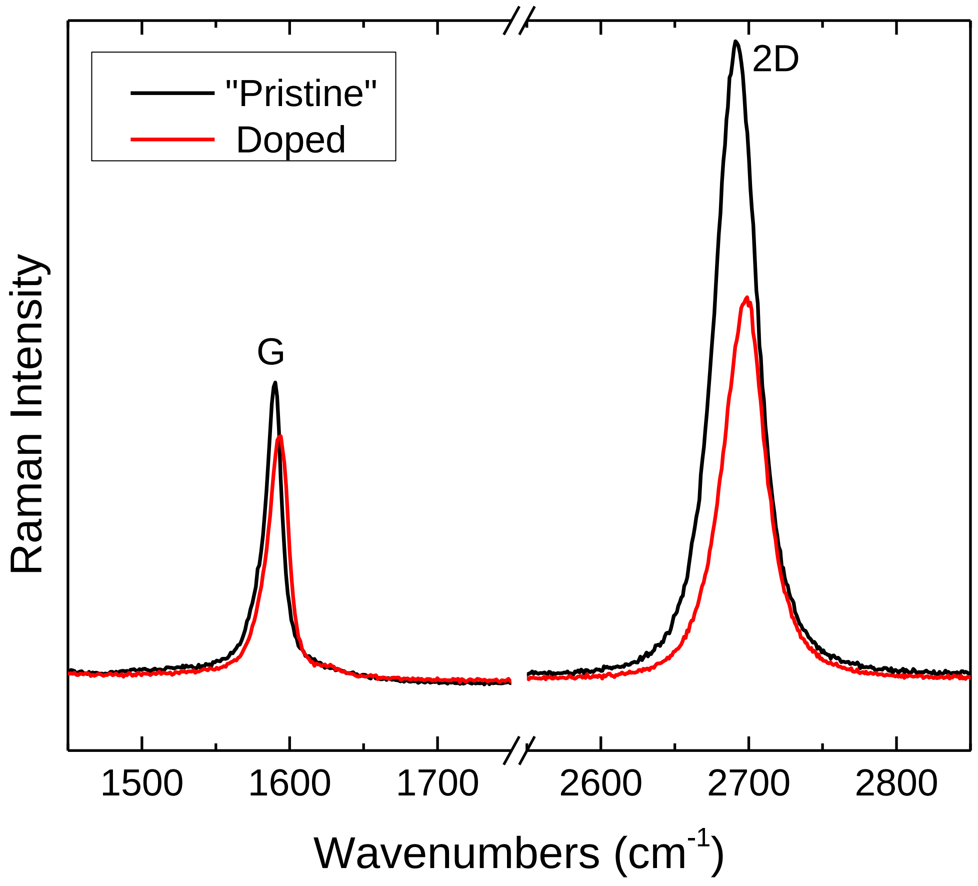

Figure S5. Characteristic Raman spectra of a CVD graphene sample in "Pristine" state and after final stage of treatment with HNO_3 vapors.
